# Digital economy, technological innovation, and sustainable development

**DOI:** 10.1371/journal.pone.0305520

**Published:** 2024-07-23

**Authors:** Wei-wei He, Shao-ling He, Hai-lan Hou

**Affiliations:** 1 School of Economics, Hunan University of Finance and Economics, Changsha, Hunan, China; 2 School of Medical Humanities and Management, Hunan University of Medicine, Huaihua, Hunan, China; 3 School of Economics and Trade, Hunan University, Changsha, Hunan, China; Inner Mongolia University, CHINA

## Abstract

This paper examines the impact of the digital economy on sustainable development, using panel data from cities at the prefecture level and above in China from 2011 to 2019. The results indicate: (1) The digital economy is conducive to boosting growth, increasing employment, reducing energy consumption, and cutting emissions, thereby promoting sustainable development. These findings prove robust. (2) Mechanism test outcomes reveal that, from the perspective of technological innovation, the digital economy can promote sustainable development through increasing R&D input and enhancing innovation output. (3) An extended analysis of the risk of a digital "divide" demonstrates that "dividend" of the digital economy is primarily manifests in spurring economic growth, enhancing energy efficiency, and strengthening environmental protection in lagging regions, while the digital "divide" effect is manifested in the stronger employment stimulating effect of developed regions versus backward areas. The results of this study not only enrich the relevant research system, but also provide empirical evidence to support accelerating digital transformation, strengthening technological innovation governance, and advancing sustainable development.

## 1. Introduction

Since the reform and opening-up period, China’s rapid urbanization movement has led to the accumulation of factor resources in modern sectors, significantly accelerating industrialization. The modernization transformation of China’s economy not only directly drives the leapfrog development of the national economy, but also helps to effectively absorb the surplus labor force, particularly from rural areas [1]. Nevertheless, it’s crucial to acknowledge that there’re still many bottlenecks to maintaining stable economic growth in China in the future. China’s modern sector-first strategy over the past four decades has led to an inefficient, high-emissions production model that is not only a diminishing engine of economic growth, but also causes significant waste of resources and numerous environmental problems that burden people’s daily lives [2]. Therefore, the need to comprehensively improve energy efficiency and reduce emissions has become a dominant concern in public discourse, particularly in terms of promoting growth and stabilizing employment. As the Chinese economy progresses to a new stage of development, the traditional growth mode based on a single path of intensive factor input has become unsustainable. Building a sustainable development model covering the core content of growth, employment, energy, and environment [3] will be the only way for China to promote economic transformation and optimization and high-quality development at this stage. But the question for China now is how to accelerate the realization of its Sustainable Development Goals. Scholars representing traditional creative destruction theory led by Schumpeter pointed out that significant societal innovations accelerate the obsolescence of older technologies and production methods, simultaneously giving rise to new production systems. Recent studies have also explored the output effects of technological innovation based on economic growth [4], labor employment [5], energy efficiency [6], and environmental pollution [7], providing a viable idea to promote sustainable development.

The 21st century has seen the emergence and widespread adoption of digital technologies, such as the Internet and 5G communications, IPv6 and cloud computing, artificial intelligence, and big data. The digital economy, with these digital technologies as its core element, has penetrated and integrated the national economy in an all-round, deep, and multifarious way, becoming a new engine of economic growth after the agricultural economy and the industrial economy. It will elevate human social productivity to a new level. Digital technology has ushered in new transformations in the content, form, and concept of production, thereby fundamentally altering the production process. Notably, the rapid development of the digital economy over recent years has fostered new space for innovation and the development of the Chinese economy. The primary impact of the digital economy on innovation manifests in three ways: improving the innovation ecosystem, exerting the back-forcing mechanism, and reducing the cost of innovation [8]. This raises a new question: Does the digital economy promote sustainable development from the perspective of technological innovation? The technological innovation effect driven by the digital economy in various dimensions, including enterprise production, financial development, industrial upgrading, and other dimensions, not only directly affects output and employment changes, but also contributes significantly to energy conservation, efficiency improvement, and emission reduction [9]. The effects of the digital economy on employment [10], energy efficiency [11], economic growth [12], and environmental pollution [13, 14] have been the subject of an increasing number of research. However, it is unfortunate that the existing research has not provided a comprehensive and profound analysis of the characteristics and evolving patterns of the digital economy and sustainable development under the tide of technological innovation. Thus, this paper attempts to evaluate the significant influence of the digital economy on sustainable development from the perspective of technological innovation by establishing an evaluation framework encompassing economic growth, labor employment, energy efficiency, and environmental pollution. By doing so, it hopes to provide a useful addition to the current research system.

Compared with the existing research, the specific potential marginal contribution of this paper may lie in two aspects: First, Compared with the existing studies, the output effect of digital economy is mainly teased from the aspects of economic growth [12], labor employment [10], energy efficiency [11] and environmental protection [13]. By constructing an analytical framework for sustainable development, this paper systematically organizes the impact of the digital economy on economic growth, employment, energy efficiency, and environmental protection. Additionally, it integrates the idea of technological innovation into the traditional information economy theory. These advancements not only enhance scientific comprehension of the digital economy but also serve to supplement and improve the current theoretical research system of information economics and development economics to some extent.

Second, by considering the typical facts of China’s digital economy in terms of its development and sustainability, this paper explores the sustainable development effect of the digital economy and its mechanism, particularly through the perspective of technological innovation. Previous research has primarily focused on the output effect of the digital economy from a single perspective of economic growth [12], labor employment [10], energy efficiency [11] and environmental protection [13]. However, there is a notable lack of studies addressing the current situation, trends, and mechanisms related to the "sustainable development effect of the digital economy from the perspective of technological innovation". This paper establishes a sustainable development index system drawing from Hou et al. (2022) [15] and measures the digital economy’s development index based on the method of Zhao et al. (2020) [12]. Furthermore, this paper empirically investigates the impact of the digital economy on sustainable development and its underlying mechanism from the perspective of opportunity equity within the dimension of technological innovation. Moreover, the impact of the digital economy’s sustained growth on the "digital divide" or "digital dividend" in a region is thoroughly examined in this study. The aforementioned research will provide empirical support for hastening the establishment of the digital economy and encouraging the creation of superior development.

The rest of the paper is structured as follows: The second part is the literature review; The third part sets up the empirical model and explains the variables. The fourth part is an empirical test and result analysis; The fifth part is the test results and analysis of the mechanism of action; The sixth part is divided into digital "divide" or "dividend": based on the regional perspective of expansion research; The seventh part is the conclusion and policy suggestion.

## 2. Literature review

As mentioned above, the effect of the digital economy on sustainable development has not been fully investigated in current research. We therefore sorted, pertinent study literature from the perspectives of economic growth, labor employment, energy efficiency, and environmental pollution based on the connotation of sustainable development.

### 2.1 Digital economy and economic growth

The growth driven by the digital economy can be attributed to the following general reasons: First, as new production factors emerge from the digital realm, such as data and traffic, the overall output is increased by optimizing the types and proportions of components. The greater and more varied the amount and type of data, the more information and knowledge will be produced. And the scale effects of data elements yield bigger economic gains when the economy is more open to its data [16]. Secondly, as digital technology permeates production processes, it actively facilitates and deepens the integration of traditional production factors such as capital, labor, and land. This integration optimizes the division of labor, improves resource utilization efficiency, and drives output expansion [17]. Then, digital financial services derived from the combination of digital technology and the financial sector can significantly reduce the financing costs of high-tech enterprises with high operating risks, large initial investments, and long R&D (Research and Experimental Development) cycles, and promote economic transformation and upgrading [18–20]. Finally, the emergence of new industries, business models, and forms driven by the digital economy (such as the sharing economy, food delivery, live streaming sales, etc.) has profoundly impacted traditional industries, accelerating the industrial iteration and economic transformation and upgrading. The integrated development of the real economy and the digital virtual economy is conducive to promoting the substitution of market capital with social capital that emphasizes interaction and emotion. This fusion amplifies the Pareto-improving effects of digital technology in the production process and encourages mutual pursuit between physical R&D and digital virtual R&D, ultimately causing a "creative destruction" to the traditional industries and market fundamentals [21].

### 2.2 Digital economy and employment

The impact of the digital economy on employment is evident in both labour demand and supply. In terms of labor demand, digital technology and resources have entered the production field, improving production efficiency and expanding the output margin of enterprises. This, in turn, increases the demand for local labor factors. Through logical deduction, it is pointed out that the widespread use of digital technology can enhance production efficiency and reduce product prices, thereby expanding social demand. This increase in social demand will further encourage manufacturers to expand production capacity, leading to increased demand for Labour [22, 23]. However, it is important to note that the digital economy can shape labor demand as well. Technological advancements driven by the digital economy are transforming the industrial structure, leading to the emergence of new industries, new business forms, and new models. Consequently, the employment prospects for low-skilled workers are undergoing significant changes, while the demand for middle- and high-skilled workers is on the rise [10].

Regarding labor supply, the digital economy can significantly reduce the cost of labor switching, thereby accelerating labor flow and increasing the scale of labor supply in modern sectors. First, the digital economy lessens the expenditure on searching employment information and enhances the likelihood of securing employment through efficient mass information compilation, screening, and precision dissemination [24]. Secondly, digital finance is conducive to loosening individual financing constraints, effectively alleviating tangible costs, including trans-regional transportation and frictional unemployment costs encountered by workers during career transition, and encouraging trans-regional and cross-sector labor flow to promote employment [25]. Finally, the digital economy can also overcome intangible costs, such as the uncertainty of new environment (encompassing geographical climate, human customs, etc.) and the loss of social capital in job conversion. By mitigating these factors, the digital economy contributes to augmenting employment [26].

### 2.3 Digital economy and energy efficiency and environmental protection

The technological innovation effect of the digital economy has significantly improved the efficiency of energy factors [27, 28]. On the one hand, digital factors have not only partially replaced other production factors but have also facilitated the integration of traditional factors, thereby improving the efficiency of energy factors. On the other hand, the collection and aggregation of green technology information on the digital platform facilitate the enhancement of market information transparency and stimulate the R&D of the production sector in the energy sector. With the improvement of energy efficiency driven by the development of digital economy, the decline in the proportion of energy input will also significantly alleviate local emission problems [3, 9, 13–14, 29, 30]. It should be emphasized that the differences in the participation capacity of the digital economy may exacerbate the "digital divide" problem, but rather affect energy efficiency [11] and unbalanced development in environmental protection efforts [31, 32].

According to the existing research, while the digital economy is beneficial for economic growth, its impact on labor employment, energy efficiency, environmental protection remains uncertain due to various constraints, such as industry characteristics, digital divide, and other factors. In China’s current stage of rapid digital economy expansion, there has been a steady increase in the scale of labor employment [5], production energy consumption [11], and gradual improvement in carbon emission efficiency [13]. This suggests that, at present, China lacks the objective conditions for the digital economy to negatively affect labor employment, energy efficiency, and environmental protection. Therefore, this paper proposes Hypothesis 1: The digital economy is conducive to promoting sustainable development.

### 2.4 Digital economy and technological innovation

Technological innovation is the fundamental driving force of of industrial transformation and upgrading, and its promoting effect on economic growth, labor employment, energy efficiency and environmental protection has been unanimously recognized by the academic community [33]. The digital economy plays a crucial role in improving the innovation ecosphere, leveraging the backward forcing mechanism, and reducing innovation costs, thereby rapidly propelling technological innovation [30, 34, 35]. First, there is a shift in production focus from producers to consumers as a result of the widespread, deep, and pervasive penetration of the digital economy into the economy and society. This also encourages the diversification of innovation subjects. Open innovation initiatives help emerging technologies reach their full commercial profitability potential [36]. Second, the emergence of new industries, business forms, and models stemming from the advancement of the digital economy motivates enterprises to extend the industrial chains and help broaden the scope of innovation. Thirdly, digital technology’s ability to collect, present, and filter massive information aids producers in accurately tracking the progression of cutting-edge technologies, thus reducing the risks in innovative activities. Fourth, under the digital wave, consumer demand for product differentiation has been further amplified, which will in turn force producers to increase R&D efforts to meet market needs. Fifth, the digital economy not only improves factor utilization rates and significantly reduces production costs but also facilitates integration with the financial sector, easing financing constraints, thereby notably decrease R&D expenditures [20].

It can be found that technological innovation is crucial to the way the digital economy influences sustainable development, yet previous research has not thoroughly addressed this topic. Therefore, hypothesis 2 is further advanced in this topic: technological innovation is the mechanism of digital economy promoting sustainable development.

## 3. Empirical model setting and variable description

### 3.1 Setting of the empirical model

To investigate the actual impact of digital economy development on sustainable development, this paper aims to construct the following basic empirical model:

$${sustainablity}_{it}=\beta_{0}+\beta_{1}{digital}_{it}+\lambda X_{it}+\mu_{i}+\delta_{t}+\varepsilon_{it}$$ (1)

In Formula (1), *sustainability*_*it*_ is the sustainable development level of *i* city in *t* year, *digital* is the development level of the digital economy, *X* is the control variable set of city at prefecture level and above, *μ* and *δ* are the fixed effect of city and fixed effect of year respectively, *ε* is the random disturbance term.

### 3.2 Variable description

#### 3.2.1 Explained variables

Sustainable development (*sustainability*). Based on the research of Hou et al. (2022) [15] and the concepts of economic growth (*growth*), employment (*employment*), energy efficiency (*energy*), and environmental protection (*co*2) related to sustainable development, this paper selects per capita GDP (unit: ten thousand yuan/person), number of employees per unit (unit: ten thousand people), and the energy intensity (ratio of standard coal consumption to GDP, unit: Ton / 10,000 yuan) and carbon emission intensity (ratio of total carbon emissions to GDP, unit: ton / 10,000 yuan). In this paper, natural gas, liquefied petroleum gas, and the electricity consumption of the whole society combined with standard coal conversion coefficient are used to convert the annual standard coal consumption at the prefecture level and above in China. The conversion formula of standard coal usage is as follows:

$$energy=\gamma E_{1}+\omega E_{2}+\tau (\eta \times E_{3})$$ (2)

*E*_1_ is the consumption of natural gas, *E*_2_ is the consumption of liquefied petroleum gas, *E*_3_ is the consumption of electricity in the whole society, *γ*, *ω* and *τ* are respectively the conversion coefficients of unit natural gas, liquefied petroleum gas and social electricity consumption, respectively, are 13.3000 tons/10,000 m^3^, 1.7143 tons/ton and 1.2290 tons/10,000 kw·h. *η* is the proportion of coal electricity in the total power generation. In terms of social electricity demand, coal dominates the current power generation structure in many regions today. This means that the use of coal contributes significantly to the carbon emissions generated by the consumption of social electricity, which provides a method to calculate carbon emissions caused by social electricity consumption. In addition, although the proportion of coal-fired power generation in different regions of China is different, it is not too great. Therefore, the calculation of coal-fired power generation in prefecture-level and above cities in China is based on the unified proportion in the China Electric Power Yearbook over the past years.

Further, the calculation formula for carbon emissions is as follows:

$$co2=C_{1}+C_{2}+C_{3}=kE_{1}+\upsilon E_{2}+\phi (\eta \times E_{3})$$ (3)

Where *C*_1_, *C*_2_ and *C*_3_ respectively represent the carbon emissions caused by natural gas, liquefied petroleum gas, and electricity consumption in the whole society, *k* is the CO_2_ conversion coefficient of natural gas, *υ* is the CO_2_ conversion coefficient of liquefied petroleum gas. *φ* is the greenhouse gas emission coefficient of the coal power fuel chain. Based on the research of He et al. (2023) [2], this paper selects the corresponding coefficient value according to the type of energy to convert the scale of carbon emissions, in which the conversion coefficient per unit of natural gas is 2.1622 kgCO_2_/m^3^, and the conversion coefficient per unit of liquefied petroleum gas is 3.1013 kgCO_2_/kg. The conversion coefficient of power consumption per unit is 1.3203 kgCO_2_/kw·h.

#### 3.2.2 Explanatory variables

Digital economy (*digital*). Given the rapid penetration of the digital economy into every field of the national economy and society, the content of the digital economy is numerous and diverse. Zhao et al. (2020) [12] research methodology offers a practical solution for gauging the development level of the digital economy in cities at the prefecture level and above in China. Therefore, based on this method, combined with two dimensions of information technology development and digital finance, this paper uses SPSS principal component analysis method to re-measure the digital economy development index of cities at the prefecture level and above in China from 2011 to 2019.

In the dimension of information technology development, this paper selects several indicators. Specifically, the number of Internet broadband access users per 100 people reflects Internet popularity. The proportion of computer service and software industry employees in urban units represents the current employment situation in the information industry. The total number of telecom businesses per capita provides insight into the output status of the information industry. Finally, the number of mobile phone users per 100 people illustrates the popularity of mobile phone.

Concerning the level of digital finance, this paper selects the China Digital Inclusive Finance Index published by Peking University as the reflection indicator of the development status of digital finance. Before the data measurement, the aforementioned indicators underwent normalized, and the specific method was: processing value = (actual value—minimum value)/(maximum value—minimum value).

The measurement results of digital economy are shown in Table 1. The KMO value is greater than 0.7, indicating that variable selection has the basic requirements for factor analysis. The component factor of the analysis results is 3, and the cumulative variance rate is about 0.89, indicating that the variable data has strong explanatory power. Further, the principal component analysis method is employed to assign weights to each analysis index, and then the initial weights are divided by the aggregate of all weights to derive the actual measurement weights for each analysis index of the digital economy.

**Table 1:** Digital economy each sub-index weight measurement results.

Primary indicators	Secondary indicators	Tertiary indicators	Weight
digital economy	information technology development	the number of Internet broadband access users per 100 people	0.24
		the proportion of computer service and software industry employees in urban units	0.17
		the total number of telecom businesses per capita	0.21
		the current situation of information the number of mobile phone users per 100 people	0.22
	digital finance	China Digital Inclusive Finance Index	0.16

#### 3.2.3 Control variables

Industrial development (*second*). Industrial scale has a direct impact on energy consumption and carbon emission levels in addition to stimulating local economic growth and employment [37]. In order to represent the level of industrial development, this paper chooses to use the the secondary industry’s added value as a percentage of GDP.

Land urbanization (*urban*). Government planning and utilization of land resources hold significant influence over the supply of local land elements, and can trigger alterations in local output [38]. Therefore, this paper selects the proportion of municipal district area in the total administrative area to reflect land urbanization.

Human capital (*hc*). Human capital accumulation is beneficial to optimize the local labor supply structure, which is very important to improve the efficiency of local output [39]. The human capital index used in this research is constructed to represent the degree of human capital in cities at the prefecture-level cities and above. The specific method is: *hc* = (number of university students ×16+ number of ordinary middle school students ×12+ number of primary school students ×6)/regional total population.

Public transport facilities. The level of public transport infrastructure has influenced economic growth, employment, environmental protection and other fields, and its promoting role in sustainable development has been gradually paid attention to by the existing research [40]. The per capita number of public vehicles (*vehicle*) is selected in this paper to reflect the level of local public transport facilities.

In addition, an increasing number of studies conducted in recent years have demonstrated that environmental regulations will not only assist limit local pollution but also help accelerate the renewal of local industries and optimize the output structure [41]. Therefore, per capita green space (*greenland*) is selected in this paper to reflect the intensity of local environmental regulations.

### 3.3 Data sources and descriptive statistics of variables

In this paper, in order to create a balanced panel data collection containing 282 cities at or above the prefecture level, samples of Chinese cities with significant data deficiency from 2011 to 2019 were removed. Based on the perspective of technological innovation, the impact of the digital economy on sustainable development and its mechanism were investigated. Among them, the digital financial inclusion index data comes from the China Digital Financial Inclusion Index published by Peking University. The rest of the data comes from the China City Statistical Yearbook. Descriptive statistics of relevant variables are shown in Table 2:

**Table 2:** Descriptive statistics of variables.

Variable	Obs	Mean	Std.	Min	Max
growth	2538	5.25	3.41	0.65	46.77
employment	2538	59.96	89.96	5.11	986.87
energy	2538	0.09	0.10	0.00	1.79
co2	2538	0.48	0.56	0.00	9.61
digital	2538	0.13	0.06	0.02	0.60
second	2538	46.94	10.62	11.70	89.34
urban	2538	0.24	0.24	0.00	1.00
hc	2538	1.33	0.54	0.46	4.97
vehicle	2538	3.86	6.92	0.10	110.52
greenland	2538	4.38	6.34	0.00	73.01

Calculated and organized by the author.

**Fig 1:**
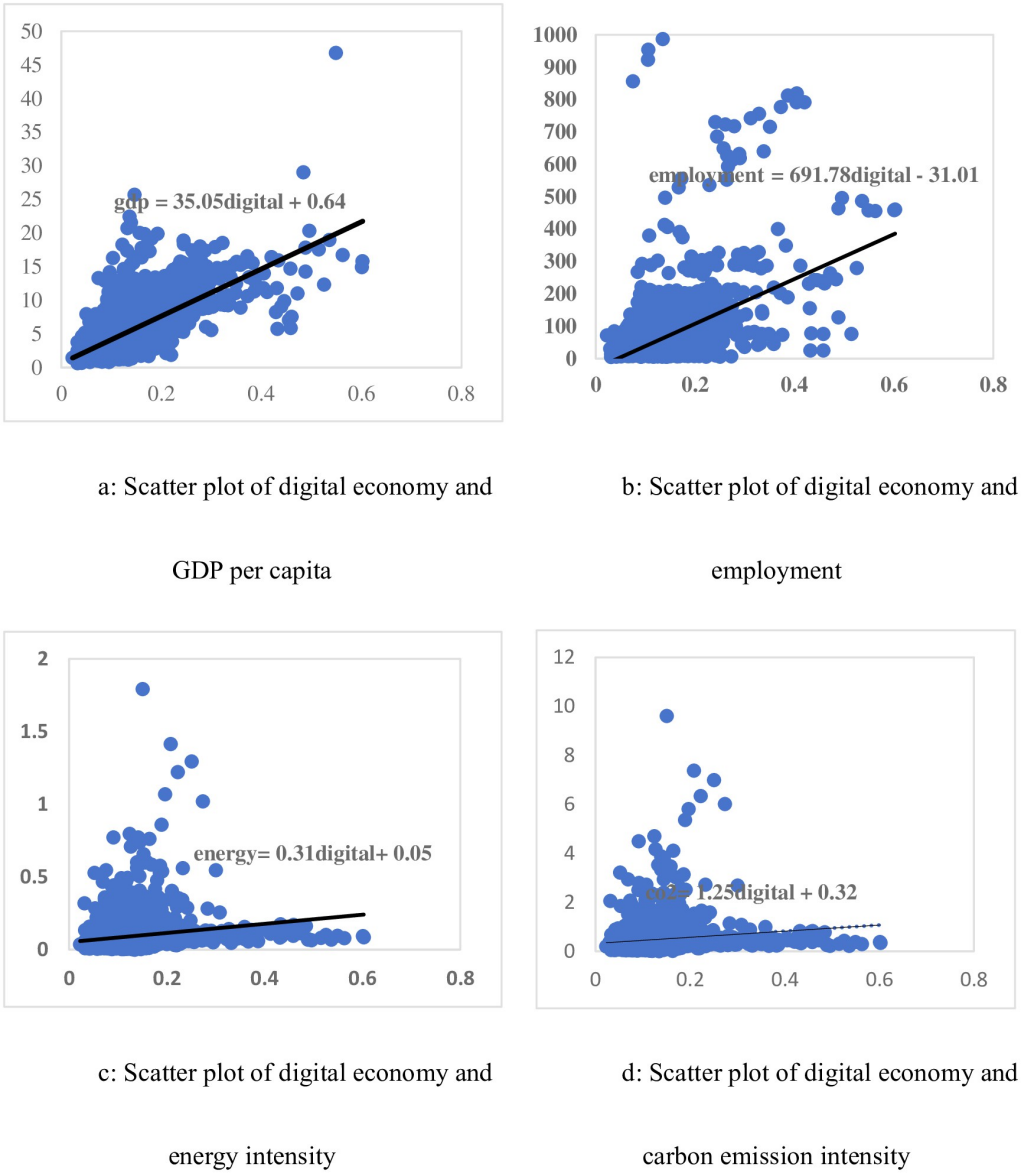
Scatter plot of digital economy and sustainable development.

A scatter plot of the sub-variables of the digital economy and sustainable development is shown in Fig 1. Based on the scatter plot and the trend line, it can be roughly concluded that the improvement of the digital economy will lead to an increase in GDP per capita, employment, energy intensity, and carbon emission intensity. In order to further investigate the actual impact of digital economy on sustainable development, this paper will build an empirical model to systematically test the sustainable development effect of digital economy.

## 4. Empirical test and result analysis

### 4.1 Basic model test

In Table 3, columns (1)—(4) show the test results of the basic model that introduces various control variables and controls the city effect and year effect. The results in columns (1)—(2) demonstrate that the digital economy has a significantly positive impact on per capita GDP and the number of employees per unit after controlling variables are introduced and the city effect and year effect are controlled,. In other words the digital economy drives regional economic growth and employment, which is consistent with the findings of Bogliacino and Pianta (10) [5]. Columns (3)—(4) show the impact of the digital economy on energy efficiency and environmental protection. It can be found that the parameters of the digital economy on energy intensity and carbon emission intensity are significantly negative, indicating that the digital economy is conducive to reducing energy consumption and carbon emission per unit of GDP, thus improving energy efficiency and alleviating environmental pollution, which is roughly the same as the research conclusions of Wang and Cao (2019) [11], Xu et al. (2022) [13].

**Table 3:** Basic model test results.

VARIABLES	growth	employment	energy	co2
	(1)	(2)	(3)	(4)
digital	11.8534***	57.9650*	-0.2177***	-0.9799***
	(1.3424)	(30.3330)	(0.0639)	(0.3425)
second	0.0893***	0.3582**	-0.0024***	-0.0138***
	(0.0067)	(0.1523)	(0.0003)	(0.0017)
urban	1.6498***	0.8643	-0.0370*	-0.1785
	(0.4548)	(10.2757)	(0.0216)	(0.1160)
hc	0.8753***	9.3833**	-0.0277***	-0.1533***
	(0.2028)	(4.5821)	(0.0096)	(0.0517)
vehicle	-0.0085	0.2384	-0.0010**	-0.0050**
	(0.0087)	(0.1977)	(0.0004)	(0.0022)
greenland	0.0068	-0.2052	0.0042***	0.0162***
	(0.0173)	(0.3907)	(0.0008)	(0.0044)
Constant	-2.0583***	22.8397**	0.2661***	1.4576***
	(0.4366)	(9.8659)	(0.0208)	(0.1114)
City effect	YES	YES	YES	YES
Year effect	YES	YES	YES	YES
Ob	2,538	2,538	2,538	2,538
R^2^	0.9057	0.9307	0.7663	0.7691

Standard errors in parentheses, *** p<0.01, ** p<0.05, * p<0.1, the same as below.

Among the control variables, the parameters for the proportion of value added by the secondary industry on per capita GDP and the number of employees per unit are significantly positive. In contrast, the parameters of energy intensity and carbon emission intensity are significantly negative. These results suggest that industrialization promotes sustainable development. The ratio of municipal area to the administrative area has a significantly positive impact on per capita GDP, a significantly negative impact on energy intensity, and an insignificant impact on the number of employees per unit and carbon emission intensity, indicating that land urbanization promotes sustainable development by driving population urbanization to promote economic growth and improve energy efficiency. The human capital index can not only promote economic growth and employment but also improve energy efficiency and reduce emissions. Therefore, it has a strong positive driving effect on sustainable development. The number of public transport vehicles per capita contributes to reducing energy intensity and carbon emission intensity, but its effects on growth and employment is limited. In other words, the role of public transport facilities in promoting sustainable development is mainly reflected in the energy and environmental dimensions. Analogously, the impact of per capita green space on energy intensity and carbon emission intensity is significantly negative, while the impact on per capita GDP and the number of employees per unit is not significant. This suggests that the influence of environmental regulations on sustainable development predominantly manifests in the energy and environmental aspects.

### 4.2 Robustness and endogeneity test

#### 4.2.1 Robustness test

Above, the sustainable development effect of the digital economy is discussed. To ensure the reliability and consistency of our findings, we conducted a comprehensive robustness test to further ascertain the real impact of the digital economy on sustainable development. The results are presented in Table 4.

**Table 4:** Robustness test.

VARIABLES	growth	employment	energy	co2
	(1)	(2)	(3)	(4)
digital	9.1599***	125.9936***	-0.4215***	-2.1076***
	(1.5722)	(32.4632)	(0.0732)	(0.3942)
Control variables	YES	YES	YES	YES
City effect	YES	YES	YES	YES
Year effect	YES	YES	YES	YES
Ob	2,256	2,256	2,256	2,256
R^2^	0.9067	0.9437	0.7829	0.7836
VARIABLES	growth	employment	energy	co2
	(5)	(6)	(7)	(8)
digital	8.6127***	61.0538***	-0.1931***	-0.8932**
	(1.2972)	(22.3026)	(0.0655)	(0.3471)
Control variables	YES	YES	YES	YES
City effect	YES	YES	YES	YES
Year effect	YES	YES	YES	YES
Province-Year effect			YES	YES
Ob	2,502	2,502	2,538	2,538
R^2^	0.7704	0.7704	0.9074	0.9326
VARIABLES	growth	employment	energy	co2
	(9)	(10)	(11)	(12)
digital	11.5887***	53.3280**	-0.9647***	-0.0001***
	(1.3590)	(21.3687)	(0.3510)	(0.0000)
Control variables	YES	YES	YES	YES
City effect	YES	YES	YES	YES
Year effect	YES	YES	YES	YES
Ob	2,502	2,502	2,502	2,502
R^2^	0.9046	0.9244	0.7704	0.7704
VARIABLES	growth	employment	energy	co2
	(13)	(14)	(15)	(16)
digital	-3.6917	-375.0261***	0.1738	0.8654
	(2.7305)	(61.3938)	(0.1308)	(0.7018)
digital^2^	33.7085***	938.9096***	-0.8489***	-4.0014***
	(5.1708)	(116.2634)	(0.2477)	(1.3290)
Control variables	YES	YES	YES	YES
City effect	YES	YES	YES	YES
Year effect	YES	YES	YES	YES
Ob	2,538	2,538	2,538	2,538
R^2^	0.9074	0.9326	0.7675	0.7700

In this paper, the original index of the current period is replaced by the lagged term of the digital economy to test the robustness of explanatory variable replacement, and the results are shown in columns (1)—(4). It can be found that the parameters of the digital economy are significantly positive in columns (1)—(2) and significantly negative in columns (3)—(4),. is consistent with the basic model. These results align with our basic model, reinforcing the validity of our initial findings.

Since economic activities change rules often have commonalities across different regions within the same province, this paper conducts a robustness test by further controlling for the province-year effect based on the basic model. The results are shown in columns (5)—(8). After controlling the provincial-year effect, it was found that the digital economy has a significantly positive impact on per capita GDP and employment, while having a significantly negative impact on energy intensity and carbon emission intensity. These results are consistent with the results of the basic model test.

A few cities have obvious advantages compared with other cities in the economic aggregate, population size, development policies, industrial base, and other aspects. The test results of the basic model may be affected by including these cities in the empirical scope. Therefore, this paper excludes the four municipalities directly under the central government of Beijing, Tianjin, Shanghai, and Chongqing, and conducts a robustness test again. The results in columns (9)—(12) indicate that the test results after the municipalities are excluded show that the digital economy contributes to an increase per capita GDP and the number of employees per unit as well as a reduction in energy intensity and carbon emission intensity. These findings align with those of the basic model.

An increasing number of studies have highlighted the significant agglomeration and network effects resulting from the development of the digital economy. The denser the population participating in the digital economy in the region, the more efficient the use of digital infrastructure becomes, leading to an agglomeration effect. In addition, as the number of participants in the digital economy increases in the region, the potential trading groups of individual participants also increase. This can help overcome the problem of information asymmetry and give rise to the network effect. Based on the above logic, the impact of the digital economy on sustainable development may have a potential non-linear tendency. Therefore, this paper introduces the quadratic term of the digital economy into the basic model to explore the possible nonlinear correlation between the digital economy and sustainable development. The results are shown in columns (13)—(16).

In Column (13), the linear term representing the digital economy demonstrates a significant effect on per capita GDP, whereas the quadratic term remains non-significant. This finding suggests that the digital economy exerts a nonlinear influence on economic growth.

In column (14), the influence of the digital economy on the number of employees per unit shows a significant "inverted U-shape", with an inflection point of approximately 0.20. This means that below this threshold, the digital economy does not favor employment, but once this threshold is crossed, it becomes conducive to employment. In fact, data from 2019 shows that the digital economy development level across all cities in China had already surpassed this threshold. Therefore, from a non-linear perspective, the digital economy still plays a role in driving economic growth and employment. In columns (15)—(16), the primary parameter of the digital economy is not significant while the second parameter is significantly negative, indicating that the digital economy has no significant nonlinear influence on energy intensity and carbon emission intensity.

#### 4.2.2 Endogeneity test

(1) Instrumental variables.Given the possibility of measurement errors, reverse causality, missing variables, and various other issues that may arise during data selection and model setup, which have the potential to introduce bias into the model results and give rise to endogeneity problems, it becomes imperative to conduct a comprehensive and systematic endogeneity test for the model. This ensures that any interference caused by the above problems does not affect the model test results. In this paper, we initially employ the instrumental variable method to refine the model, and the relevant test results are shown in Table 5.

**Table 5:** Endogeneity test results.

VARIABLES	growth	employment	energy	co2
	(1)	(2)	(3)	(4)
digital	75.6245***	1,473.3485***	-2.8081***	-12.8909***
	(14.4323)	(317.1981)	(0.6283)	(3.1903)
Control variables	YES	YES	YES	YES
City effect	YES	YES	YES	YES
Year effect	YES	YES	YES	YES
Kleibergen-Paap rk LM statistic	45.027	45.027	45.027	45.027
	(0.0000)	(0.0000)	(0.0000)	(0.0000)
Kleibergen-Paap rk Wald F statistic	40.494	40.494	40.494	40.494
	(16.38)	(16.38)	(16.38)	(16.38)
Ob	2,538	2,538	2,538	2,538
R^2^	-0.4934	-0.9222	-0.5291	-0.4729

The test results processed using the instrumental variable method are presented in column (1). This paper selects the historical data of the number of fixed-line telephone subscribers in 2004 as the instrumental variable for the digital economy development index, aiming to address the potential endogenous problems inherent in the basic model. The early development of the Internet is closely tied to the number of local fixed telephone subscribers, which is why this indicator was chosen. This is because early Internet development frequently depended on fixed telephone connectivity. Additionally, the number of fixed phone users in various regions is trending downward year over year due to the widespread development of mobile phone services and the installation of network base stations. The historical scale of fixed phone users had little bearing on the sustainable development of the current period. The study uses the product of the year and the number of fixed telephone users in cities of various tiers and above in 2004 as the instrumental variable for the regional digital economy index because the primary data for the chosen instrumental variables constitute sectional data, making them unsuitable for direct application in the endogeneity test analysis of panel data.

Based on the findings presented in column (1), for the test of the original hypothesis of "insufficient identification of instrumental variables", the Kleibergen-Paap rk LM test yielded a statistical p-value of 0.00, which falls below the critical threshold of 0.01. Consequently, the original hypothesis is significantly rejected. In the test of weak recognition of instrumental variables, the Kleibergen-Paap rk Wald F statistic is about 41.152, larger than the critical value at the 10% level of the Stock-Yogo weak recognition test. The aforementioned results demonstrate the appropriateness of utilizing the product of the number of fixed telephone users in each city and the corresponding year as the instrumental variable for the development index of the digital economy. In the model test outcomes obtained through the application of these instrumental variables, the digital economy parameters are significantly positive. This indicates that the digital economy, as processed through these instrumental variables, exerts a driving effect on sustainable development, aligning with the findings observed in the basic model.

(2) DID. On August 17, 2013, the General Office of the Ministry of Housing and Urban-Rural Development issued the Notice on the Application of the 2013 National Smart City Pilot Project. This notice selected cities at the prefecture level and above to participate in the national Smart City pilot project, which was implemented in three batches across 2013, 2014, 2015. The initiative aimed to expedite urban construction and development through the utilization of modern information technology.

In fact, with the implementation of the Smart City pilot, the relevant pilot cities will make use of emerging digital technologies such as 5G communication and the Internet of Things, big data and cloud computing, artificial intelligence, and blockchain. These technologies foster a social atmosphere that encourages innovation and development, further driving the optimization and adjustment of local production modes. The Smart City pilot has greatly improved the scale of local digital users, network speed, coverage, and integration degree with economic and social development. This not only reinforces the hardware infrastructure construction for the local digital economy development but also establishes an excellent experimental environment for analyzing exogenous policy impacts. In this paper, the multi-phase difference (DID) method was employed to assess how the "Smart City" pilot affected sustainable development:

$${sustainability}_{it}=\beta_{0}+\beta_{1}{did}_{it}+\lambda D_{it}+\mu_{i}+\delta_{t}+\varepsilon_{it}$$ (4)

Where *i* represents the city and *t* represents the year. did indicate whether the city was the pilot city of a "Smart City" in the current year. If yes, it is 1; otherwise, it is 0. The results of the difference-difference test are shown in Table 6.

**Table 6:** Results of DID test.

VARIABLES	growth	employment_	energy	co2
	(1)	(2)	(3)	(4)
did	0.1721***	7.6221***	-0.0155***	-0.0795***
	(0.0587)	(2.0150)	(0.0043)	(0.0277)
Control variables	YES	YES	YES	YES
City effect	YES	YES	YES	YES
Year effect	YES	YES	YES	YES
Ob	2,538	2,538	2,538	2,538
R^2^	0.9492	0.9310	0.7664	0.7691

Columns (1)—(4) exhibit the outcomes of DID model. The parameter demonstrated a statistically significant positive in columns (1)—(2), whereas it exhibited a significantly negative in columns (3)—(4). This suggests that the exogenous policy influence had a significant promoting effect on economic growth, labor employment, energy efficiency, and environmental protection. Therefore, taking into account the test results of DID model, it can be concluded that the "smart city" pilot does drive sustainable development.

(3) propensity matching score. In this paper, the vernier caliper propensity matching score model with a 1% degree of freedom was chosen to conduct the endogeneity test in order to further overcome potential endogeneity problems of the model. Table 7 displays the balance trend test findings for various variables. Following matching, all variables had standardized differences of less than 10%, indicating that each variable is well-balanced. In addition, the T-test results after each variable matching did not reject the original hypothesis of systematic differences between the treatment group and the control group. Therefore, it can be considered that the basic model setting and data selection in this paper is suitable for the propensity matching score test.

**Table 7:** Results of the equilibrium trend test.

VARIABLES	Before and after matching	Experimental group	Control group	Difference	t -value	p-value
second	U	46.56	47.24	-6.40	-1.58	0.11
	M	46.60	46.80	-1.90	-0.41	0.68
urban	U	0.27	0.22	22.10	5.57	0.00
	M	0.26	0.26	-0.20	-0.04	0.97
hc	U	1.44	1.24	37.00	9.45	0.00
	M	1.41	1.41	-1.60	-0.37	0.71
vehicle	U	5.09	2.90	31.10	8.00	0.00
	M	4.30	4.35	-0.70	-0.22	0.83
greenland	U	5.57	3.45	32.90	8.48	0.00
	M	4.89	4.69	3.10	0.87	0.39

The results of the propensity matching score test are shown in Table 8 below. It can be found that ATT parameters in columns (1)—(2) are significantly positive, indicating that smart city pilot is beneficial to employment and economic growth. The ATT parameters in columns (3)—(4) are significantly negative, indicating that smart city pilots can also effectively reduce carbon emissions and energy consumption.

**Table 8:** Propensity matching scores.

VARIABLES	growth	employment	energy	co2
	(1)	(2)	(3)	(4)
ATT	1.5338***	27.8956***	-0.0163**	-0.1032***
	(0.1343)	(4.4489)	(0.0070)	(0.0370)
ATU	1.0358***	18.8746***	-0.0115***	-0.0837***
	(0.1319)	(2.8148)	(0.0036)	(0.0194)
ATE	1.2531***	22.8109***	-0.0136***	-0.0922***
	(0.1251)	(3.1816)	(0.0046)	(0.0244)
Ob	2,538	2,538	2,538	2,538

Drawing from the results of the basic model, robustness, and endogeneity tests, it can be concluded that the digital economy significantly promotes sustainable development, Thus, Hypothesis 1 can be proved.

#### 4.2.3 Heterogeneity analysis: Based on the perspective of internet development and digital finance

Based on the aforementioned measurement process of the digital economy index, it can be found that Internet development and digital finance are the two main components driving the digital economy development in China’s prefecture-level and above cities. As digital infrastructure progresses comprehensively and digital inclusive finance evolves,the question remains: what are the practical impacts of the Internet and digital finance on sustainable development, and how do they differ from each other? The above problems await further resolution. Therefore, based on the perspectives of Internet development and digital finance, this paper examines the impact of sustainable development of the digital economy, which is of great importance to describe the impact of the digital economy on sustainable development in an in-depth and comprehensive way. Relevant results are shown in Table 9.

**Table 9:** Results of the heterogeneity test.

VARIABLES	growth	employment	energy	co2
	(1)	(2)	(3)	(4)
internet	8.7055***	48.9036*	-0.1155*	-0.5034
	(1.2789)	(28.7054)	(0.0605)	(0.3245)
Control variables	YES	YES	YES	YES
City effect	YES	YES	YES	YES
Year effect	YES	YES	YES	YES
Ob	2,538	2,538	2,538	2,538
R^2^	0.9044	0.9307	0.7654	0.7685
VARIABLES	gdp	employment	energy	co2
	(1)	(2)	(3)	(4)
finance	5.0009***	13.5042	-0.1626***	-0.7534***
	(0.6570)	(14.7908)	(0.0310)	(0.1664)
Control variables	YES	YES	YES	YES
City effect	YES	YES	YES	YES
Year effect	YES	YES	YES	YES
Ob	2,538	2,538	2,538	2,538
R^2^	0.9049	0.9306	0.7679	0.7703

In this paper, we normalize the digital finance index published by Peking University is normalized to assess the sustainable development effect of the development of digital finance. The relevant results, presented in columns (5)—(8), indicate that can be found that digital finance has a significantly positive effect on per capita GDP, albeit with a smaller parameter value compared to Internet development. Put differently, while digital finance has a positive role in economic growth, it does not have the same impact as good as that of Internet development. The influence of digital finance on the employment scale is not significant, meaning that digital finance has no obvious promoting effect on employment. Furthermore, digital finance has a strong negative impact on energy intensity and carbon intensity, indicating that it can advance environmental protection as weel as energy efficiency.

In conclusion, it is evident that in driving the sustainable process of the digital economy, Internet development contributes more to expanding the positive output dimension, whereas digital finance plays a greater role in elevating factor efficiency and reducing the negative output dimension.

## 5. Mechanism inspection and analysis

Combined with the aforementioned comprehensive and systematic demonstration, the propelling effect of the digital economy on sustainable development has been confirmed. Considering the digital economy’s vital role in improving the innovation ecosystem, activating forcing mechanism, and reducing the cost of innovation, it becomes imperative to delve into the mechanism by which the digital economy impacts sustainable development, particularly from the perspective of technological innovation. This paper aims to construct a mediation effect model to discuss the mechanism of the digital economy promoting sustainable development. Specifically, utilizing the basic empirical model as a foundation and adopting a technological innovation lens, we establish a linear correlation model of intermediary variables and a mechanism model introducing intermediary variables. To investigate whether "innovation input" and "innovation output" play a substantial mediating role in the process of the digital economy affecting sustainable development. The details are as follows:

$${inter}_{it}=\beta_{0}+\beta_{1}{digital}_{it}+\lambda D_{it}+\mu_{i}+\delta_{t}+\varepsilon_{it}$$

$${sustainability}_{it}=\beta_{0}+\beta_{1}{digital}_{it}+\beta_{2}{inter}_{it}+\lambda D_{it}+\mu_{i}+\delta_{t}+\varepsilon_{it}$$ (5)

Based on the perspective of "innovation input", the R&D intensity reflects innovation input (*rd*), which is measured by the proportion of local science and technology expenditure in GDP. Table 10 presents pertinent test findings. The parameters of the digital economy in column (1) are significantly positive, indicating that the R&D intensity has improved greatly with the growth of the digital economy, this is consistent with the conclusions of Chen et al. (2023) [34], Ding et al. (2024) [30], Han et al. (2024) [35] and other studies. The results in columns (2)—(3) show that the parameters of the digital economy and R&D intensity are both positive and significant, implying that R&D intensity plays a mediating role in the process of the digital economy promoting economic growth and employment. According to the results in columns (4)—(5), both parameters of the digital economy and R&D intensity are significantly negative. This suggests that, R&D intensity serves as an intermediary factor in the process by which the digital economy contributes to the reduction of energy consumption and carbon emissions.

**Table 10:** Results of mechanism test: From the perspective of innovation input.

VARIABLES	rd	growth	employment	energy	co2
	(1)	(2)	(3)	(4)	(5)
digital	0.0043***	11.4184***	48.9621	-0.2096***	-0.9363***
	(0.0012)	(1.3403)	(30.3068)	(0.0640)	(0.3432)
rd		101.0445***	2,090.8183***	-1.8667*	-10.1164*
		(22.5864)	(510.7372)	(1.0786)	(5.7841)
Control variables	YES	YES	YES	YES	YES
City effect	YES	YES	YES	YES	YES
Year effect	YES	YES	YES	YES	YES
Ob	2,538	2,538	2,538	2,538	2,538
R^2^	0.8070	0.9065	0.9312	0.7666	0.7694

Next, this paper uses the number of patents per capita as an indicator of innovation output (*innovation*), examining the role of technological innovation in promoting sustainable development within the digital economy. The number of patents per capita is expressed by the number of patent applications per 10,000 people. The relevant test results are shown in Table 11. The parameters of the digital economy in column (1) are significantly positive, indicating that the development of the digital economy favorably impacts the augmentation of patents per capita. The results in columns (2)—(3) reveal that the parameters of per capita patent number are significantly positive, implying that the digital economy assumes an intermediary function in stimulating growth and employment. The results in columns (4)—(5) show that the parameters of the per capita patent number are significantly negative. This suggests that the number of patents per capita serves as an intermediary factor in the process of reducing energy consumption and carbon emissions within the digital economy.

**Table 11:** Results of mechanism test: From the perspective of innovation output.

VARIABLES	innovation	growth	employment	energy	co2
	(1)	(2)	(3)	(4)	(5)
digital	45.6786***	6.5194***	28.8754	-0.1879***	-0.8054**
	(6.6708)	(1.1049)	(30.3532)	(0.0644)	(0.3452)
innovation		0.1168***	0.6368***	-0.0007***	-0.0038***
		(0.0035)	(0.0951)	(0.0002)	(0.0011)
Control variables	YES	YES	YES	YES	YES
City effect	YES	YES	YES	YES	YES
Year effect	YES	YES	YES	YES	YES
Ob	2,538	2,538	2,538	2,538	2,538
R^2^	0.8631	0.9374	0.9320	0.7673	0.7703

Base on the aforementioned research results, it is evident that technological innovation does play a pivotal role in promoting sustainable development, aligning with the study conducted by Cai et al.(2023) [33]. Furthermore, the positive driving effect of digital economy on technological innovation is also highly consistent with the research conclusions of Chen et al. (2023) [34], Ding et al. (2024) [30], Han et al. (2024) [35]. Additionally, the test results of the intermediary model demonstrate that technological innovation serves as a crucial mechanistic factor in driving the sustainable progress of the digital economy, thereby validating hypothesis 2.

## 6. "Digital divide" or "digital dividend": An expanded analysis based on a regional perspective

It is worth emphasizing that the sustainable development effect of the digital economy also contingent upon the development level of the digital economy across distinct regions. In other words, disparities in the digital economy’s levels can directly result in regional differences in sustainable progress. This has the potential to cause distortions in the regional market, exacerbate imbalances in regional growth, and ultimately impede the overall sustainable progress of the economy. If the sustainable development effect of backward regions is stronger under the background of digital construction being rolled out in an all-around way, it will form an obvious digital universal benefit feature to fully release the "digital dividend". Conversely, if developed regions’ impact on sustainable development outweighs that of backward regions, the "digital dividend" of developed regions would further expanded and ultimately transform into the "digital divide" between regions.Upon sorting out the existing research, it can be found that the regional digital divide primarily stems from the digital infrastructure divide, digital terminal divide, digital software divide, and digital capability divide.

First of all, the unbalanced regional allocation of digital infrastructure encompassing elements such as 5G communication base stations, big data centers, smart cities, and rail transit, results in disparities in the public’s basic prerequisites for engaging in the digital economy in different regions. This variance further widening the inter-regional welfare divide, and ultimately forming the "digital infrastructure gap". Secondly, the quantity of terminal devices like computers, smartphones, and tablet computers. Which serve as the medium for individuals to engage in digital activities, directly affects the local digital economy’s capacity for intervention. Considering the currently high cost of relevant digital terminal equipment, local digital participation capability is also closely linked to the local capacity and willingness to pay (such as the level of economic development, income, consumption, and consumer preference). Disparities in payment capacity and willingness will restrict the purchase of digital terminal equipment in backward regions, thereby widening the "digital terminal equipment gap" among regions. leading to variations in the welfare effects of the digital economy among different regions [42]. Furthermore, digital software is the participation path of the digital economy. Nevertheless, the functional development of market-oriented and application-oriented software struggles to cover the diverse needs of all users. Software manufacturers, whose strategies often prioritize large markets, may have to overlook the utilization needs of comparatively smaller or "minority" groups. This tendency can potentially exacerbate the "digital software gap" between regions, thereby amplifying development differentiation. Finally, the application of digital terminals and digital software is intricately tied to individual’s ability to use digital technology. Differences in the ability to use digital technology between residents in different regions give rise to a "digital ability gap". Evidently, the development of the digital economy appears to be a "double-edged sword". Hence, it remains a matter of debate whether digital economy will widen social inequalities or pay dividends in the terms of promoting sustainable development. To address these questions, this paper conducts an in-depth analysis of the sustainable impact of the digital economy from the perspective of the regional "digital divide". In this paper, according to the digital economy level and above of cities in China in 2011, the sample cities are divided into four groups from low level to high level with 25%, 50%, and 75% as sub-points, and the "gap" and "dividend" of the sustainable development effect of the digital economy are empirically discussed.

### 6.1 Economic growth and employment

The test results concerning economic growth and labor employment in the context of the digital economy, viewed through the perspective of the "digital divide", are shown in Table 12. Columns (1)—(4) demonstrate that the promotion effect of the digital economy on economic growth is significant in all groups, with a greater impact on groups at higher levels compared to those at low levels. It should be pointed out that the average per capita GDP of the lower-level group rose from 27,300 yuan in 2011 to 44,800 yuan in 2019, marking an increase of approximately 64.10%. From 2011 to 2019, the average per capita GDP of the high-level group climbed from 62,200 yuan to 99,900 yuan, an increase of 59.16%. These findings indicate that, under the digital wave, the lower-level group, despite having a smaller economic growth scale, exhibited a higher growth rate. In other words, the backward region has achieved a "relative catch-up" in economic growth.

**Table 12:** Test results of the impact of the digital divide on economic growth and employment.

VARIABLES	growth	growth	growth	growth
	(1)	(2)	(3)	(4)
digital	6.1539***	4.5349**	8.8715***	12.2858***
	(1.5271)	(1.8119)	(2.0352)	(2.9237)
Control variables	YES	YES	YES	YES
City effect	YES	YES	YES	YES
Year effect	YES	YES	YES	YES
Ob	639	630	630	639
R^2^	0.9459	0.9312	0.9221	0.8524
VARIABLES	employment	employment	employment	employment
	(5)	(6)	(7)	(8)
digital	-119.0088**	-33.4922	1.2855	83.2821
	(59.6870)	(22.0226)	(28.2993)	(72.7926)
Control variables	YES	YES	YES	YES
City effect	YES	YES	YES	YES
Year effect	YES	YES	YES	YES
Ob	639	630	630	639
R^2^	0.6949	0.9279	0.9417	0.9237

The results in columns (5)—(8) reveal that the impact of the digital economy on the employment scale is significantly negative in the low-level group, while it does not have a significant effect in the other groups. This suggests that the development of the digital economy has widened the "employment gap" between regions. The potential reason behind this could be that the digital economy’s ability to collect, sort, and filter vast amounts of employment information has considerably reduced the information asymmetric in the job employment market. This acceleration in the optimal allocation of labor force elements across the country may expedite the migration of surplus labor from underdeveloped areas to developed areas, resulting in further differentiation of regional labor employment patterns.

### 6.2 Energy efficiency and environmental protection

Table 13 displays test findings for energy efficiency and environmental protection of the digital economy from the perspective of the "digital divide". The energy efficiency test results are shown in columns (1)—(4). It can be found that the digital economy has no significant influence on the energy intensity of the low-level group and the high-level group, while the influence on the low-level group and the high-level group is significantly negative. Additionally, the low-level group’s absolute value of parameter values is greater than the high-level group’s. Put differently, the digital economy contributes more to enhancing energy efficiency in backward regions.

**Table 13:** Test results of the impact of the digital divide on energy efficiency and environmental protection.

VARIABLES	energy	energy	energy	energy
	(1)	(2)	(3)	(4)
digital	-0.1659**	-0.1913	-0.1624**	-0.0526
	(0.0810)	(0.1301)	(0.0797)	(0.0538)
Control variables	YES	YES	YES	YES
City effect	YES	YES	YES	YES
Year effect	YES	YES	YES	YES
Ob	639	630	630	639
R^2^	0.8604	0.7118	0.8776	0.8283
VARIABLES	co2	co2	co2	co2
	(5)	(6)	(7)	(8)
digital	-0.7400*	-1.2646*	-0.9045**	-0.0769
	(0.4229)	(0.6656)	(0.4475)	(0.2695)
Control variables	YES	YES	YES	YES
City effect	YES	YES	YES	YES
Year effect	YES	YES	YES	YES
Ob	639	630	630	639
R^2^	0.8470	0.7391	0.8546	0.7939

The environmental protection inspection results are shown in columns (5)—(8). It can be seen that the digital economy has no significant impact on carbon emission intensity in the high-level group, but is significant in the other groups. Specifically, the digital economy promotes environmental protection in the backward areas while having a limited effect on the environment in the advanced areas.

Drawing from the aforementioned conclusions, it becomes evident that the characteristics of the digital economy’s sustainable development impact are relatively intricate in the context of the "digital divide." On one hand, the digital economy has delivered substantial "digital dividends" in terms of economic growth, energy efficiency, and environmental protection. On the other hand, it has also exacerbated the "digital divide" in the realm of labor and employment.

## 7. Conclusions and policy recommendations

### 7.1 Main conclusions

Since the 1980s, China has experienced rapid economic growth fueled by industrialization and urbanization, significantly enhancing the welfare of its citizens.

However, in recent years, due to the sustained slowdown in marginal growth, the conflict between economic growth and resources and environment, sustainable development, as well as the tension between pursuing short-term profits and long-term sustainable development, has become increasingly prominent. This issue has garnered widespread attention from various sectors of economic society. With the leapfrog development of the digital technology revolution, digitization has penetrated the entire national economy in an all-around, deep, and wide field, opening up new avenues for accelerating economic transformation and propelling high-quality development. From the perspective of technological innovation, this paper systematically answers the problem of realizing sustainable development under the digital wave and seeks to provide empirical support for strengthening the construction of the digital economy and promoting sustainable development.

First, this paper conducts a comprehensive and systematic review of extant research, highlighting that the digital economy can stimulate economic growth, expand employment, enhance energy efficiency, and foster environmental protection through the encouragement of technological innovation, thereby attaining sustainable development. Second, this paper employs the panel data from 282 cities at or above the prefecture level in China, spanning from 2011 to 2019, to conduct empirical tests. The findings reveal a statistically significant and robust positive impact of digital economy development on sustainable progress. Additional heterogeneity tests indicate that as two major components of the digital economy, Internet development contributes more to the expansion of the positive output dimension, whereas digital finance demonstrates a more pronounced effect in improving factor efficiency and reducing the negative output dimension. Thirdly, from the lens of technological innovation, this paper performs a mechanistic analysis on the effect of digital economy development on sustainable development, focusing on two aspects of R&D input and R&D output. The results illustrate that expanding R&D intensity and enhancing innovation activity constitute a pivotal avenue for the digital economy to promote sustainable development. Fourthly, upon regional investigation of whether the sustainable development driven by the digital economy’s development constitutes a "gap" or a "bonus", it is found that the digital economy has unleashed a "digital dividend" in the dimensions of economic growth, energy efficiency, and environmental protection, yet it has widened the "digital divide" in the labor and employment sphere.

### 7.2 Policy inspiration

1. Comprehensively accelerate the development of the digital economy. First, increase investment in digital infrastructure construction, with a special focus on bolstering the infrastructure in regions where the digital economy lags, thereby improving the supply level of digital public facilities. Second, foster the development process of digital industrialization, aggressively expanding the scale of the digital industry. Establish and construct several demonstration zones encompassing digital equipment manufacturing, information services, e-commerce, and other allied industries. Facilitate the refinement and realignment of the industrial layout, while intensifying research endeavors in core technologies such as high-end chips, industrial software, and operating systems. Third, leverage advanced digital technologies such as 5G communications, artificial intelligence, big data analytics, cloud computing, blockchain, and more, to enhance the functionality of digital software and increase the accessibility and convenience of participating in the digital economy. Fourth, it is necessary to broaden the scope of digital technology training to effectively enhance individuals’ proficiency in engaging with the digital economy, thereby empowering them with the necessary skills.
2. Strengthen the coordinated governance capacity for sustainable development of the digital economy. Firstly, it is necessary to continually foster the integration of data and reality, specifically focusing on facilitating the digital transformation of traditional, high-polluting, and energy-intensive industries. This will enhance production efficiency and optimize resource utilization. Secondly, it is necessary to deepen the development of financial technology, improve the management ability of the financial investment sector in the fields of object identification, model innovation, and risk control. This will ease individuals’ access to financing and lower associated costs, ultimately optimizing capital allocation efficiency in the market. Finally, the governance process of using the digital economy to carry out sustainable economic development should be fully integrated with local industrial development, regional planning, human resources, public services, environmental regulations, And other realities, so as to build an integrated governance system for the local digital economy and sustainable development.
3. Take technological innovation as the starting point, and accelerate the digital economy and sustainable development by increasing R&D input and expanding R&D output. First, we must continuously ramp up investments in science and technology to guarantee the normal operation of technological innovation activities. Second, it’s crucial to leverage the fundamental role of market mechanisms in allocating innovation factors, bolster intellectual property protection, and refine the environment conducive to innovation research and development. Third, we must stimulate and integrate diverse components of innovation, vigorously encourage "mass innovation", school-enterprise cooperation, cross-border collaboration, transnational collaboration, and other avenues to comprehensively spearhead the development of technological innovation.

Based on the lens of technological innovation, this paper systematically discusses the effect and mechanisms of the digital economy’s sustainable development. Several conclusions are drawn through rigorous analysis, paving the way for numerous future research directions. First of all, as research in related fields continues to intensify, the measurement methodologies for indicators like the digital economy and sustainable development are destined to undergo refinement. Consequently, the research perspectives and conclusions of related issues may change significantly. Furthermore, as data availability increases, the research dimension will gradually shift from the macro to the micro perspective. Finally, compared with the rapid development of the digital economy, the theoretical research on the logic, connotation and function of the digital economy remains inadequate and requires further enhancement and refinement.
